# Coexistence of Maturity-Onset Diabetes of the Young Type 3 (MODY 3) and Diabetic Ketoacidosis: A Report of a Rare Case

**DOI:** 10.7759/cureus.96360

**Published:** 2025-11-08

**Authors:** Daniel E Ekwueme, Kyi Sann, Mohammad M Rahman

**Affiliations:** 1 Internal Medicine, Wrexham Maelor Hospital, Wrexham, GBR; 2 Diabetes and Endocrinology, Wrexham Maelor Hospital, Wrexham, GBR

**Keywords:** diabetic ketoacidosis (dka), genetic testing, hnf1a mutation, maturity-onset diabetes of the young (mody), mody 3

## Abstract

Maturity-onset diabetes of the young (MODY) is a monogenic form of diabetes often misclassified as type 1 or type 2 diabetes. We report a young woman initially diagnosed with type 1 diabetes following gestational hyperglycaemia. Autoimmune testing was negative, and genetic analysis confirmed an *HNF1A* mutation consistent with maturity-onset diabetes of the young type 3 (MODY 3). Despite sulfonylurea therapy, she required insulin and experienced recurrent diabetic ketoacidosis (DKA) due to poor adherence. Improved engagement and continuous glucose monitoring led to marked glycaemic improvement. This case highlights the diagnostic challenge of MODY presenting with DKA, a rare occurrence in this condition. Early genetic testing is essential for accurate diagnosis, personalised management, and family counselling.

## Introduction

Maturity-onset diabetes of the young (MODY) represents a heterogeneous spectrum of monogenic diabetes syndromes that are both clinically and genetically distinct. The condition typically manifests before the age of 25 years and follows an autosomal dominant inheritance pattern [1]. In contrast to type 1 diabetes, which is characterised by autoimmune beta-cell destruction, and type 2 diabetes, which is largely associated with insulin resistance and environmental factors, MODY arises from pathogenic variants in genes that regulate beta-cell function, insulin secretion, or glucose sensing [2,3]. Despite these defining features, misclassification as either type 1 or type 2 diabetes remains common, particularly in primary care, often resulting in inappropriate management and missed opportunities for genetic counselling [4].

More than 14 MODY subtypes have been described to date, each associated with specific mutations in genes central to glucose homeostasis. Among these, maturity-onset diabetes of the young type 3 (MODY 3) caused by mutations in the hepatocyte nuclear factor 1-alpha (HNF1A) gene is the most frequent form, accounting for 30-70% of genetically confirmed cases depending on population and diagnostic strategies [5,6]. Although MODY accounts for only 1-2% of all diabetes diagnoses, a substantial proportion estimated at 80 90% remains unrecognised or is inaccurately classified as type 1 or type 2 diabetes [4,6]. Individuals with MODY 3 typically demonstrate a progressive reduction in endogenous insulin production but exhibit marked sensitivity to sulfonylureas, which can provide effective glycaemic control at low doses in the early stages of the disease [6].

Accurate recognition and genetic confirmation of MODY have important clinical and familial implications. Establishing the correct molecular diagnosis facilitates personalised treatment decisions and enables predictive genetic testing in relatives at risk, supporting earlier detection and intervention [7]. Moreover, the identification of monogenic diabetes subtypes refines the taxonomy of diabetes and reflects the growing integration of precision medicine into endocrinology. With the increasing accessibility of next-generation sequencing technologies, systematic testing for MODY is expected to improve diagnostic accuracy, minimise misclassification, and optimise outcomes through genotype-informed therapeutic strategies [3,7].

## Case presentation

A female patient presented with gestational diabetes mellitus at the age of 17, which was effectively managed through dietary modification and lifestyle intervention. Following delivery, however, persistent hyperglycaemia was observed in the postnatal period. At 23, she was diagnosed with type 1 diabetes mellitus on the basis of significant hyperglycaemia and biochemical ketosis, and a basal bolus insulin regimen was initiated. Autoimmune serology revealed an atypical profile: glutamic acid decarboxylase (GAD) antibodies and islet cell antibodies were both negative, while insulin autoantibodies were markedly elevated at >5 units. The absence of GAD and islet cell antibodies does not support a typical presentation of type 1 diabetes, and this discordance prompted further consideration of a monogenic aetiology, particularly maturity-onset diabetes of the young.

Comprehensive analysis of all coding regions and exon-intron boundaries of the monogenic diabetes genes (GCK, HNF1A, HNF4A, HNF1B, NEUROD1, INS, INSR, KCNJ11, ABCC8, PDX1, PAX6, GATA6, LMNA, and PPARG) and the mitochondrial m.3243A>G (MIDD) mutation was performed using targeted next-generation sequencing (Agilent custom capture v5/Illumina HiSeq). This assay also detects partial and whole gene deletions or duplications. Exon 6 of the HNF1A gene was further analyzed by Sanger sequencing to confirm variant calls or improve coverage.

Genetic testing subsequently identified Gene: HNF1A Exon: 2 Nucleotide Change: c.476G>A Protein Change: p.Arg159Gln (p.R159Q) Consequence: Missense variant, a pathogenic variant in the HNF1A gene, confirming a diagnosis of MODY 3. In light of this result, insulin therapy was discontinued and replaced with oral sulfonylurea treatment (gliclazide). However, monotherapy with sulfonylureas failed to achieve adequate glycaemic control, necessitating the reintroduction of insulin. The patient’s family history was notable for type 2 diabetes mellitus in her maternal grandfather. Following current recommendations for the management of monogenic diabetes, her children were referred for cascade genetic screening.

Following her confirmed diagnosis, the patient experienced recurrent episodes of diabetic ketoacidosis (DKA), all attributable to suboptimal adherence to insulin therapy. Between 2015 and 2024, she required hospital admission on seven separate occasions for DKA. Each episode was marked by profound metabolic decompensation, including severe hyperglycaemia, ketonaemia, and acidosis, as illustrated in Table 1.

**Table 1:** Trend in biochemical parameters recorded at the time of each diabetic ketoacidosis (DKA) admission between 2015 and 2024. DKA Diagnostic Criteria: Hyperglycemia: Blood glucose > 11 mmol/L; Ketonemia/Ketonuria: Blood ketones ≥ 3.0 mmol/L or moderate to large urine ketones; Metabolic acidosis: Arterial pH < 7.3 and/or bicarbonate < 15 mmol/L

Date	pH	Glucose on Admission (mmol/L) (Reference Range 2.8-11.0 mmol/L)	Ketone Level (mmol/L) (Reference Range <0.6 mmol/L)
18 Nov 2015	6.99	28.7	5.2
26 Sep 2016	7.07	21.2	4.0
19 Mar 2018	6.93	25.8	6.0
18 Jun 2018	6.99	38.5	4.5
3 Jan 2019	7.31	18.7	4.1
26 Oct 2021	6.90	25.1	>7.0
13 Oct 2024	7.14	12.2	3.8

**Figure 1:**
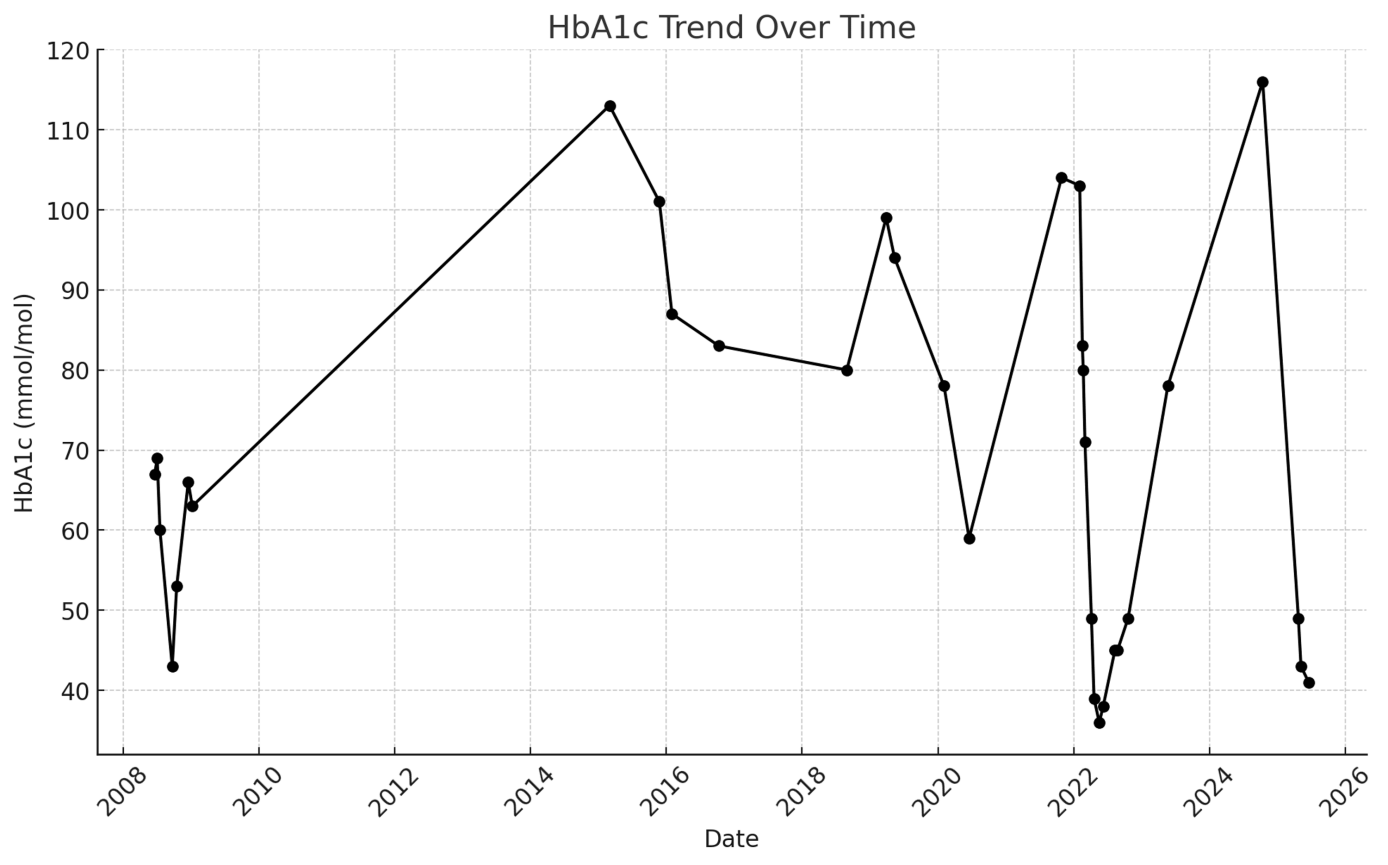
Longitudinal trend of the patient’s HbA1c levels over time. HbA1c: Glycated haemoglobin

Her glycated haemoglobin (HbA1c) levels demonstrated considerable variability over time, reflecting periods of suboptimal glycaemic control. This is shown in Figure 1, which depicts the longitudinal trend in HbA1c values throughout the course of her management.

At present, the patient is managed with a basal-bolus insulin regimen, comprising NovoRapid 10-12 units administered three times daily and Lantus 12 units at night. With renewed engagement and improved adherence, her glycaemic control has improved significantly. Her most recent HbA1c has decreased to 41 mmol/mol, compared with a previous peak of 116 mmol/mol. Continuous glucose monitoring via the FreeStyle Libre system indicates a mean glucose level of 6.7 mmol/L, with 72% of readings within the target range (4.5-8.0 mmol/L), 21% above range, and 3% below range. No values were recorded in the very high glucose range, and episodes of symptomatic hypoglycaemia have become infrequent. Despite recent improvements in metabolic control, the patient has developed background diabetic retinopathy, likely reflecting the cumulative impact of long-standing glycaemic variability and recurrent episodes of metabolic decompensation.

## Discussion

This case demonstrates the diagnostic complexity and clinical overlap between monogenic and autoimmune forms of diabetes, particularly in young adults. The initial presentation of gestational diabetes at age 17, followed by persistent postnatal hyperglycaemia and a presumed diagnosis of T1DM in early adulthood, is a pattern not uncommonly seen. However, the subsequent identification of a pathogenic variant in the HNF1A gene established the diagnosis of MODY 3. This subtype of MODY is associated with a progressive decline in insulin secretion and heightened sensitivity to sulfonylureas, often allowing effective management without insulin, particularly in the early stages [6].

A key point of distinction in this case is the development of recurrent DKA, a complication rarely associated with MODY. DKA is a hallmark of T1DM, where absolute insulin deficiency results in unopposed lipolysis and ketogenesis. In contrast, MODY subtypes, including MODY 3, typically preserve some degree of endogenous insulin production, rendering the risk of DKA exceedingly low under normal physiological circumstances [1,6]. Therefore, the seven documented DKA admissions over a nine-year period in this patient are notable and were likely precipitated by poor adherence to insulin therapy, as well as intrinsic insulin deficiency in pancreatic beta cells, an atypical finding of MODY.

This case further underscores the diagnostic challenge posed by MODY, particularly in individuals initially labelled as having T1DM. The misclassification rate is substantial: studies have shown that up to 80% of MODY cases are initially misdiagnosed, most commonly as type 1 or type 2 diabetes [4]. Shields et al. reported that fewer than 5% of patients with MODY are correctly identified at diagnosis, with the remainder inappropriately treated-often with insulin in the absence of autoimmune markers [4]. In this context, the presence of ketosis and elevated insulin autoantibodies may have confounded the diagnostic process. While insulin autoantibodies can be suggestive of T1DM, they may also be elevated in individuals recently initiated on exogenous insulin, thereby reducing their specificity in such cases [2].

The rarity of this case lies not only in the coexistence of MODY 3 and DKA, but also in the sustained pattern of DKA recurrence, which is highly atypical for MODY. This suggests that while genetic diagnosis may guide optimal treatment, long-term outcomes remain highly dependent on patient adherence and broader psychosocial factors. It also reinforces the need for ongoing education, behavioural support, and structured follow-up in managing young adults with diabetes, particularly those with complex or overlapping phenotypes.

## Conclusions

This case highlights the need to consider MODY in young patients with diabetes that does not follow the typical features of type 1 or type 2 diabetes. The patient was first treated as having type 1 diabetes and developed recurrent DKA, which is rare in MODY 3 but more common in type 1 diabetes. Genetic analysis later confirmed an HNF1A mutation, establishing MODY 3 and allowing more appropriate treatment and counselling. The unusual presentation with DKA likely reflected both reduced adherence to therapy and underlying β-cell dysfunction, showing how psychosocial and biological factors can interact to worsen glycaemic control. As genetic testing becomes more available, early recognition of MODY is essential to guide targeted management, reduce unnecessary insulin use, and support screening of affected relatives.
